# Analysis of the epidemiological characteristics of posterior malleolus fracture in adults

**DOI:** 10.1186/s13018-023-04007-w

**Published:** 2023-07-18

**Authors:** Yongqi Li, Rui Luo, Bing Li, Jiang Xia, Haichao Zhou, Hui Huang, Yunfeng Yang

**Affiliations:** 1 Department of Orthopedics, Tongji Hospital, School of Medicine, Tongji University, Shanghai, 200065 China; 2 Department of Orthopedics, Karamay Central Hospital, Karamay, 834000 China; 3 Department of Neurology, Karamay Central Hospital, Karamay, 834000 China

**Keywords:** Posterior malleolus fracture, Epidemiology, Injury mechanism, Classification

## Abstract

**Background:**

This study explores the latest epidemiological characteristics of posterior malleolus fracture and compares the epidemiological differences of posterior malleolus fracture in different periods, regions, and adult age groups.

**Methods:**

Clinical information of inpatients with posterior malleolus fracture in Shanghai Tongji Hospital and Karamay Central Hospital from January 2014 to February 2022 was reviewed and collected. The imaging data of patients were acquired using the Picture Archiving and Communication Systems. A statistical analysis was performed as to gender, current age, year of admission, injury mechanism, fracture type, and posterior malleolus fracture classification. Moreover, a comparative analysis was conducted on the injury mechanisms and morphological differences of posterior malleolus fracture at different periods, regions, and age groups.

**Results:**

A total of 472 patients (210 patients from Shanghai Tongji Hospital and 262 patients from Karamay Central Hospital) with posterior malleolus fracture and an average age of 48.7 ± 15.6 were included in this study. The peak of posterior malleolus fracture occurs in the age group of 50–59. The injury mechanisms mainly involve low-energy fall and sprain (411 cases, 87.1%), followed by traffic accidents (52 cases, 11.0%), and fall injury from height (9 cases, 1.9%). With aging, the number of fall and sprain cases increases and reaches the peak at the age of 50–59, followed by progressive decline. Traffic accidents presents a relatively flat small peak in the age group of 40–59. The number of cases according to different fracture types shows the following ascending order: trimalleolar fracture-supination external rotation (335 cases, 71.0%) > bimalleolar fracture (60 cases, 12.7%) > trimalleolar fracture-pronation extorsion (43 cases, 9.1%) > posterior malleolus + tibial shaft fracture (19 cases, 4.0%) > simple posterior malleolus fracture (15 cases, 3.2%). The numbers of cases corresponding to the Haraguchi I Type, II Type, and III Type of posterior malleolus fractures were 369 (78.2%), 49 (10.4%), and 54 (11.4%), respectively. The Tongji IIA Type represented the highest number of cases (249 cases, 52.8%), followed by the IIB Type (120 cases, 25.4%), I Type (54 cases, 11.4%), IIIB Type (36 cases, 7.6%), and IIIA type (13 cases, 2.8%). The trimalleolar fracture-supination external rotation, Haraguchi I Type and Tongji IIA Type of posterior malleolus fractures all presented an obvious peak of incidence in the age group of 50–59. However, no obvious statistical difference was observed in the injury mechanism, Haraguchi classification, and Tongji classification of posterior malleolus fractures among different years and regions in recent years (*P* > 0.05).

**Conclusions:**

The injury mechanism of posterior malleolus fracture mainly involves low-energy fall and sprain cases. The trimalleolar fracture-supination external rotation, Haraguchi I type and Tongji IIA type of posterior malleolus fracture are predilection fracture types, and all present an obvious incidence peak in the age group of 50–59. Elderly patients have high risks of falling and their bones are more fragile, conditions which are potential risk factors of posterior malleolus fracture. Early positive control has important significance. This study provides references for relevant basic and clinical studies of posterior malleolus fracture.

## Background

Posterior malleolus fracture is a common fracture type and accounts for approximately 7%–44% of ankle joint injuries [1–3]. This fracture type is caused by rotation and (or) axial violence and is often accompanied by internal and lateral malleolus fractures or ligamentous injuries. The posterior malleolus is an important bone structure to maintain ankle joint stability. If ankle joint injuries combined with posterior malleolus fracture are not treated promptly or properly, they can readily cause traumatic arthritis, thus affecting the quality of life of patients [4, 5].

Recently, the diagnosis and treatment of posterior malleolus fracture have attracted wide attention, a development which have achieved many results. However, the treatment and prognosis of posterior malleolus fracture remains controversial [6–8]. Many scholars hope to disclose the injury mechanism, damage mode, and severity through posterior malleolus fracture classification, thereby guiding treatment and judging prognosis. The Haraguchi classification [9] is the first and predominant posterior malleolus fracture classification. However, this classification is mainly based on the morphological features of CT scanning fracture and has limited significance for treatment schemes and prognosis evaluation. Subsequently, the classification system has been improved and derived continuously. The Bartonicek [10], Mangnus [11], Mason [12], and Tongji [13] classifications have been developed successively. Specifically, the current author believes that the Tongji classification elaborated upon the pathological anatomic features of posterior malleolus fracture relatively comprehensively by combining ligament structure, morphology, and injury mechanism. The Tongji classification is a relatively ideal scheme for current guidance on clinical diagnosis and treatment.

Given the poor prognosis of ankle joint damages combined with posterior malleolus fracture, in-depth study of the epidemiology, injury mechanism, and clinical and imaging features involved is vital. Such exploration is crucial to increase the control level and improve clinical effects. Nevertheless, large sample-sized epidemiological surveys of posterior malleolus fracture are rare. In this work, the data of inpatients with posterior malleolus fracture in Shanghai Tongji Hospital and Karamay Central Hospital from January 2014 to February 2022 were reviewed and investigated. The research purposes were as follows: 1) scientifically and objectively reflect the epidemiological status and development trend of posterior malleolus fracture; 2) analyze and compare the injury mechanisms and morphological differences of posterior malleolus fracture in the early (2014–2018) and late (2019–2022) stages of the research, as well as the differences between Eastern and Western China as represented by the Shanghai Tongji Hospital and Karamay Central Hospital; and 3) provide references to clinicians in the prevention, diagnosis, treatment, and relevant studies of posterior malleolus fracture.

## Methods

### Inclusion and exclusion criteria

Inclusion criteria: 1. posterior malleolus fracture; 2. Age ≥ 18; 3. Complete follow-up visit data and at least 12 months of follow-up visits.

Exclusion criteria: 1. Old posterior malleolus fracture; 2. Pathological posterior malleolus fracture; 3. Open posterior malleolus fracture.

### General information

**Fig. 1:**
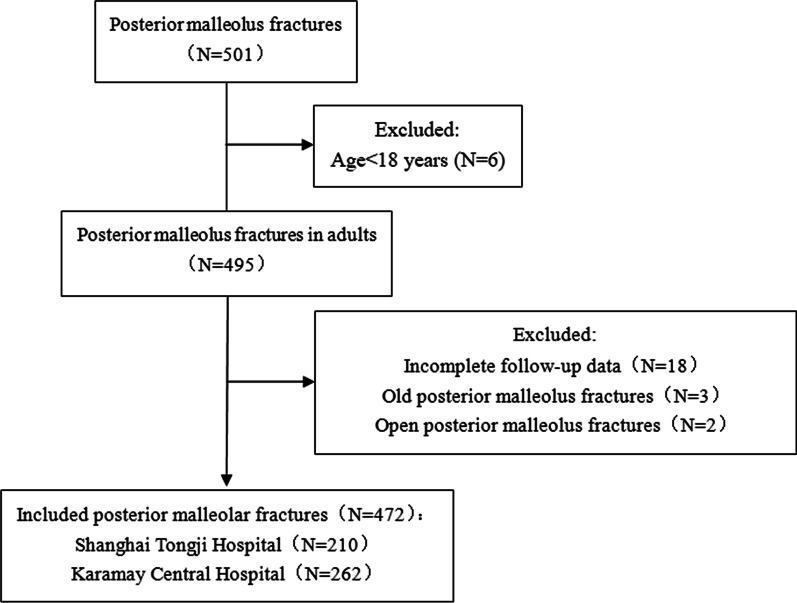
Flowchart of patient selection

Clinical information of inpatients with posterior malleolus fracture in the Shanghai Tongji Hospital and Karamay Central Hospital from January 2014 to February 2022 was collected. Further, the imaging data (X-ray and CT images of ankle joints at injury) of patients were acquired by using the Picture Archiving and Communication Systems. The data included their gender, age, year of admission,, injury mechanism, fracture type, and posterior malleolus fracture classification. A total of 501 cases of posterior malleolus fracture was identified. Among them, 472 cases were included according to inclusion and exclusion criteria, including 210 cases from Shanghai Tongji Hospital and 262 cases from Karamay Central Hospital (Fig. 1). This study was approved by the Medical Ethics Committee of Tongji Hospital, Tongji University (Grant No. K-W-2021-015).

### Grouping

All patients were divided into two groups according to the year of admission: patients from 2014 to 2018 as the early group, and those from 2019 to 2022 as the late group. To further study the epidemiological characteristics of different age groups, patients were further divided into 7 age groups at an interval of 10 years: 18–29, 30–39, 40–49, 50–59, 60–69, 70–79 and ≥ 80 years old.

### Observation indexes

#### Injury mechanism

According to electronic medical records, the injury mechanism of patients mainly consists of three types: fall and sprain, traffic accidents, and fall injury from height.

#### Comorbidities

Comorbidities refer to other internal medicine diseases in patients with posterior malleolus fracture, such as hypertension, diabetes, cerebrovascular disease, coronary heart disease, and arrhythmia, etc.

#### Fracture type

Analysis of the image data of patients reveal 5 types of fractures in this study: 1. Pure posterior malleolus fracture (P); 2. Bimalleolar fracture (posterior malleolus + lateral malleolus/fibular fracture) (PL); 3. Trimalleolar fracture-supination external rotation (posterior malleolus + lateral malleolus/fibular + medial malleolus fracture/ deltoid ligament injury) (PLM-SER); 4. trimalleolar fracture-pronation external rotation (posterior malleolus + lateral malleolus/fibular + medial malleolus fracture/ deltoid ligament injury-pronation external rotation) (PLM-PER); and 5. posterior malleolus + tibial shaft fracture (PT).

#### Haraguchi classification of posterior malleolus fracture [9]

Haraguchi classification is the first posterior malleolus fracture classification proposed and covers three types: I Type: posterior lateral oblique fracture and posterolateral wedge fracture of the distal tibia; II Type: the internal extension type. The fracture line extends to the malleolus medialis transversely. This type of fracture is also called the posterior Pilon fracture; III Type: microshell type and lamellar fracture at posterior distal tibia.

#### Tongji classification of posterior malleolus fracture [13]

Combining the posterior malleolus ligament structure as well as the morphological features and injury mechanism of posterior malleolus fracture blocks, the Tongji classification covers 5 subtypes under 3 types: I Type: fracture in the tibial attachment zone of the inferior transverse tibiofibular ligament; II Type: fracture in the tibial attachment zone of the inferior transverse tibiofibular ligament and posterior inferior tibiofibular ligament, which can be divided into IIA and IIB Subtypes according to whether the posterior malleolus fracture is accompanied by cartilago articularis and subchondral bone injuries, compression, or die-punch bone blocks; III Type: fracture in the tibial attachment zone of the inferior transverse tibiofibular ligament, posterior inferior tibiofibular ligament, and posterior tibiotalar ligament, which can be divided into Subtypes IIIA and IIIB according to number of posterior malleolus fragments.

### Statistical analysis

The normality test of data was carried out using SPSS 25. 0 and the Kolmogorov–Smirnov method. The measurement data (age) in normal distribution were expressed by x ± s. Enumeration data were expressed by the number of cases, and inter-group comparison (injury mechanism) employed the Chi-square test. The Haraguchi and Tongji classifications of posterior malleolus fracture were viewed as ranked data, and the Kruskal–Wallis H test was applied. All tests were bilateral. P < 0.05 indicates statistical significance of differences.

## Results

### Demographic characteristics

**Fig. 2:**
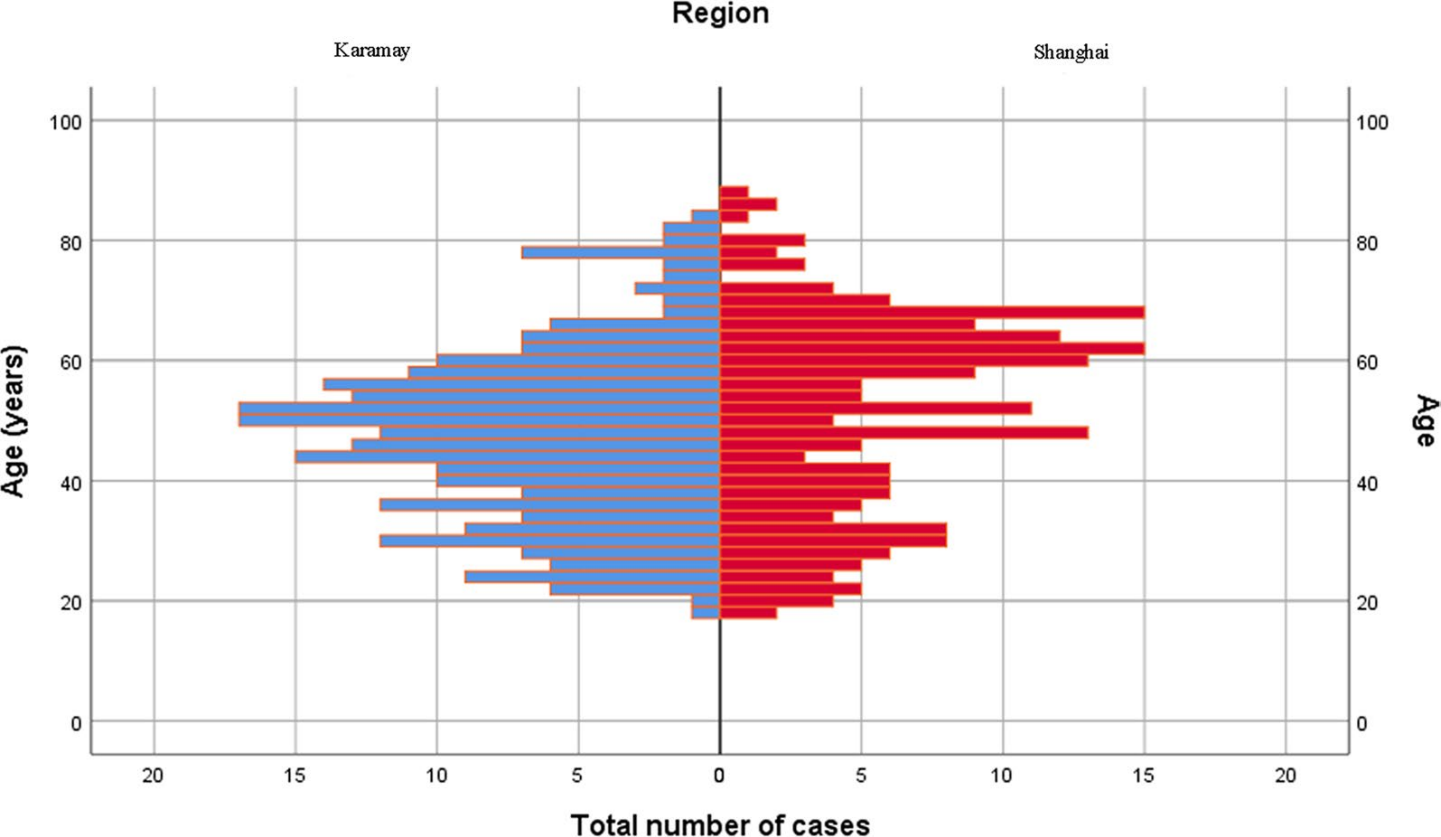
Age distribution of posterior malleolus fracture

This study involved 472 patients with posterior malleolus fracture, aged 18–88 years, and averaging at (48.7 ± 15.6) years. Patients in the 50–59 age group presented the peak number of posterior malleolus fracture cases in the Shanghai Tongji Hospital and Karamay Central Hospital (Fig. 2). Among the 472 patients, 220 were male (46.6%), aged 18–84, with an average of (43.3 ± 14.9) years. Conversely, there were 252 female patients (53.4%), aged 18–88, with an average of (53.4 ± 14.7) years. The male to female ratio was 1:1.15.

### Comorbidities

In this study, 145 patients had comorbidities (30.7%), and 327 patients had none (69.3%). The main comorbidities include diabetes, hypertension, and coronary heart disease, followed by viral hepatitis, bronchial asthma, and mental diseases (Tables 1 and 2).

**Table 1:** Epidemiological characteristics of patients with posterior malleolus fracture

	Overall (N = 472)	Male (N = 220)	Female (N = 252)
Region			
Karamay	262 (55.5%)	129 (58.6%)	133 (52.8%)
Shanghai	210 (44.5%)	91 (41.4%)	119 (47.2%)
Comorbidities			
Yes	145 (30.7%)	53 (24.1%)	92 (36.5%)
No	327 (69.3%)	167 (75.9%)	160 (63.5%)
Injury mechanism			
Fall and sprain	411 (87.1%)	182 (82.7%)	229 (90.9%)
Traffic accidents	52 (11.0%)	30 (13.6%)	22 (8.7%)
Fall injury from height	9 (1.9%)	8 (3.6%)	1 (0.4%)
Fracture type^*^			
P	15 (3.2%)	10 (4.5%)	5 (2%)
PL	60 (12.7%)	33 (15%)	27 (10.7%)
PLM-SER	335 (71.0%)	138 (62.7%)	197 (78.2%)
PLM-PER	43 (9.1%)	25 (11.4%)	18 (7.1%)
PT	19 (4.0%)	14 (6.4%)	5 (2%)
Haraguchi classification			
I	369 (78.2%)	163 (74.1%)	206 (87.1%)
II	49 (10.4%)	22 (10%)	27 (10.7%)
III	54 (11.4%)	35 (15.9%)	19 (7.5%)
Tongji classification			
I	54 (11.4%)	35 (15.9%)	19 (7.5%)
IIA	249 (52.8%)	109 (49.5%)	140 (55.6%)
IIB	120 (25.4%)	54 (24.5%)	66 (26.2%)
IIIA	13 (2.8%)	7 (3.2%)	6 (2.4%)
IIIB	36 (7.6%)	15 (6.8%)	21 (8.3%)

^*^P, Pure posterior malleolus fracture; PL, Bimalleolar fracture (posterior malleolus + lateral malleolus/fibular fracture); PLM-SER, Trimalleolar fracture-supination external rotation (posterior malleolus + lateral malleolus/fibular + medial malleolus fracture/ deltoid ligament injury); PLM-PER, trimalleolar fracture-pronation external rotation (posterior malleolus + lateral malleolus/fibular + medial malleolus fracture/ deltoid ligament injury); PT, posterior malleolus + tibial shaft fracture

**Table 2:** Comorbidity distribution of patients

Comorbidities		Number of cases	Percentage (%)
Simple hypertension		7	4.8
Simple diabetes		15	10.3
Simple coronary heart disease		7	4.8
Hypertension + coronary heart disease		25	17.2
Hypertension + diabetes		24	16.6
Hypertension + diabetes + coronary heart disease		25	17.2
Bronchial asthma		5	3.4
Viral hepatitis		6	4.1
Epilepsy		2	1.4
Hyperuricemia		4	2.8
Cerebral infarction		5	3.4
Renal insufficiency		3	2.1
Anemia		5	3.4
Hypothyroidism		2	1.4
Psychosis: depression,anxiety disorder		6	4.1
Psoriasis		2	1.4
Malignant tumors of gastrointestinal tract		1	0.7
Pregnancy		1	0.7
Total		145	100

### Injury mechanism

**Fig. 3:**
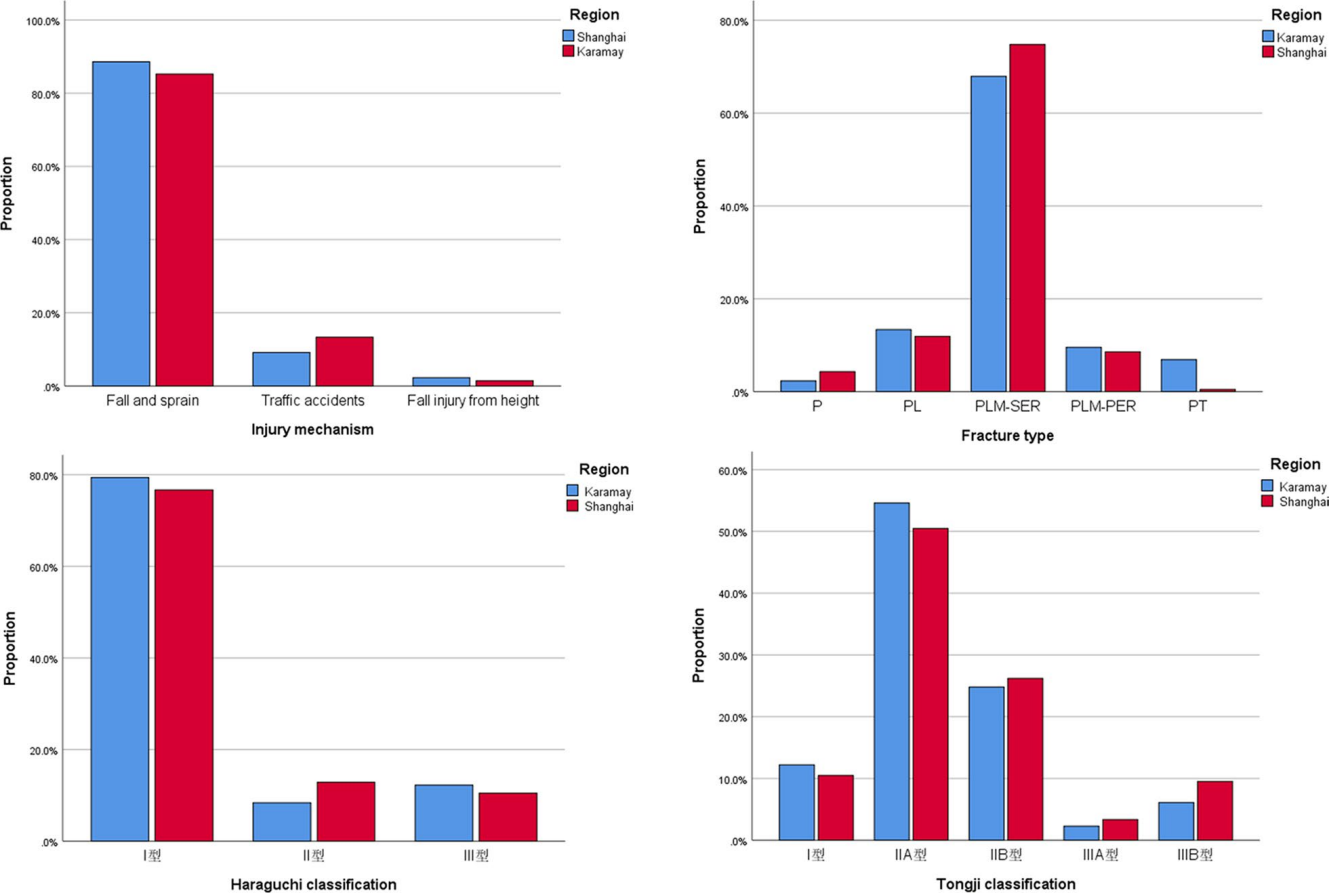
Distribution pattern of injury mechanism, fracture type, Haraguchi classification, and Tongji classification of posterior malleolus fracture

**Fig. 4:**
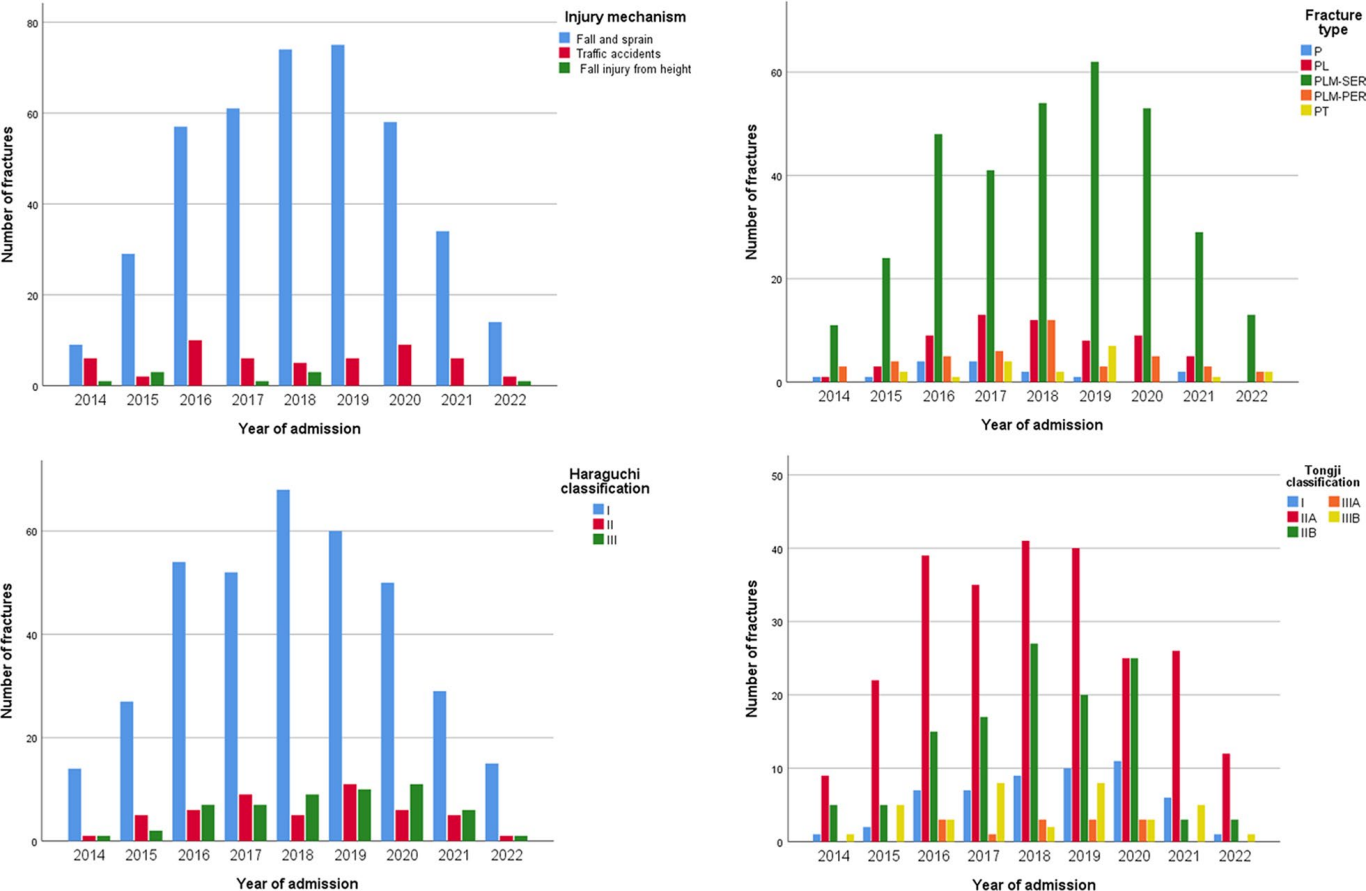
Distribution pattern of injury mechanism, fracture type, Haraguchi classification, and Tongji classification of posterior malleolus fracture with time

**Fig. 5:**
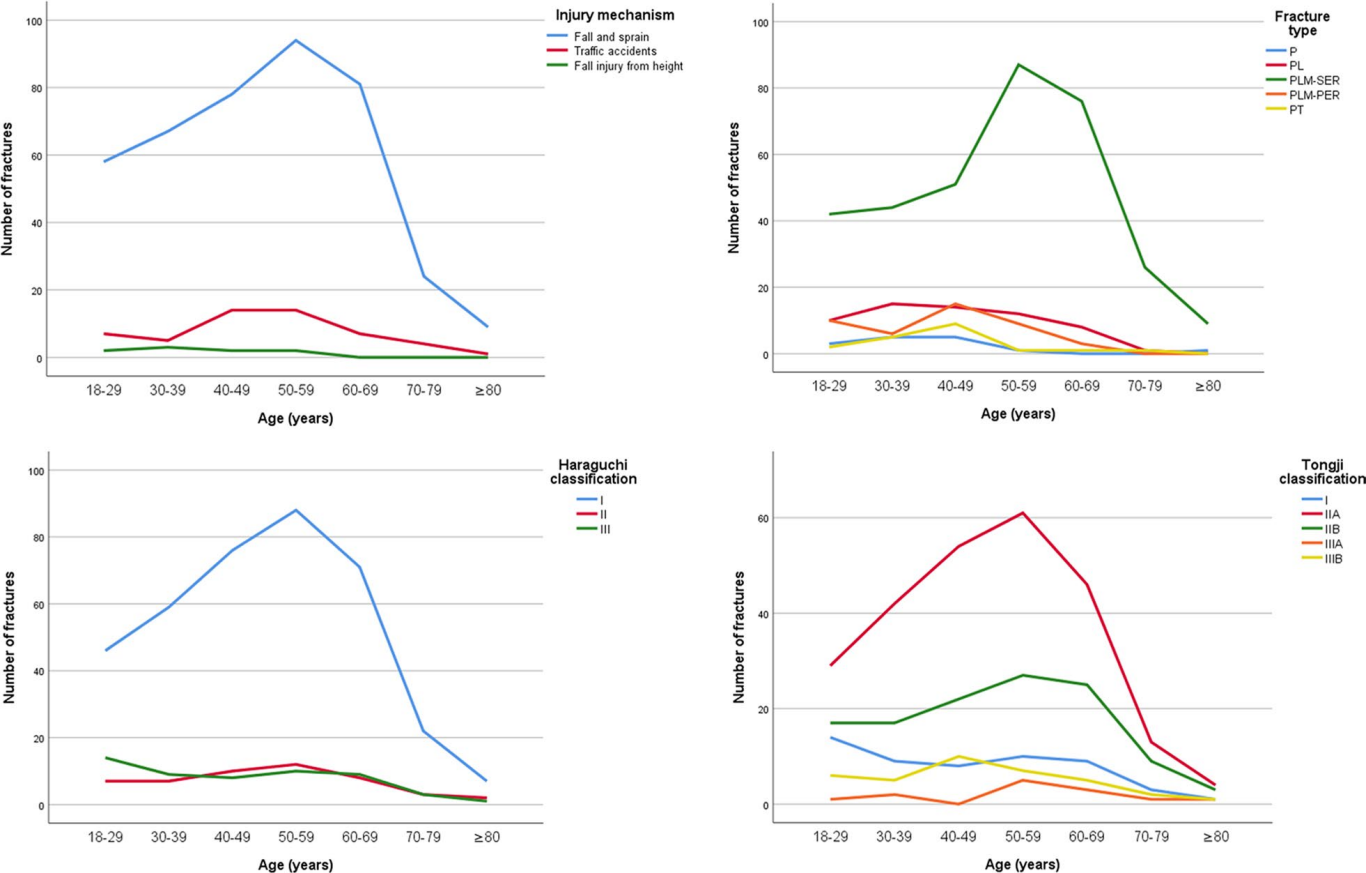
Distribution pattern of injury mechanism, fracture type, Haraguchi classification, and Tongji classification of posterior malleolus fracture with age

The injury mechanism involved in this work mainly entails low-energy fall and sprain, followed by traffic accidents, and fall injury from height (Table 1 and Fig. 3). However, no statistically significant differences were observed between the two hospitals as regards the injury mechanism of posterior malleolus fracture (X^2^ = 2.4, P = 0.295) (Table 3 and Fig. 3). Moreover, Table 4 and Fig. 4 indicate no obvious change in the major injury mechanism at different years (X^2^ = 4.0, P = 0.135). The curves in Fig. 5 indicate that with the increase of age, the cases of fall and sprain increases gradually and reaches a peak in the 50–59 age group, followed by a progressive decrease. Traffic accidents present a relatively flat peak in the 40–59 age group. However, fall injury from height has a low incidence rate in all age groups.

**Table 3:** Comparison between Shanghai Tongji Hospital and Karamay Central Hospital patients in terms of the injury mechanism, Haraguchi classification, and Tongji classification of posterior malleolus fracture

	Karamay (N = 262)	Shanghai (N = 210)	X^2^/H	*P*
Injury mechanism				
Fall and sprain	232 (88.5%)	179 (85.2%)	2.4	0.295
Traffic accidents	24 (9.2%)	28 (13.3%)		
Fall injury from height	6 (2.3%)	3 (1.4%)		
Haraguchi classification				
I	208 (79.4%)	161 (76.6%)	0.3	0.598
II	22 (8.4%)	27 (12.9%)		
III	32 (12.2%)	22 (10.5%)		
Tongji classification				
I	32 (12.2%)	22 (10.5%)	2.2	0.136
IIA	143 (54.6%)	106 (50.5%)		
IIB	65 (24.8%)	55 (26.2%)		
IIIA	6 (2.3%)	7 (3.3%)		
IIIB	16 (6.1%)	20 (9.5%)

**Table 4:** Comparison of the injury mechanism, Haraguchi classification, and Tongji classification of posterior malleolus fracture between the early (2014–2018) and late (2019–2022) stages of the study period

	Early stage (N = 267)	Late stage (N = 205)	X^2^/H	*P*
Injury mechanism				
Fall and sprain	231 (86.5%)	180 (87.8%)	4.0	0.135
Traffic accidents	28 (10.5%)	24 (11.7%)		
Fall injury from height	8 (3.0%)	1 (0.5%)		
Haraguchi classification				
I	215 (80.5%)	154 (75.1%)	2.3	0.132
II	27 (10.1%)	22 (10.7%)		
III	25 (9.4%)	29 (14.1%)		
Tongji classification				
I	25 (9.4%)	29 (14.1%)	0.3	0.616
IIA	147 (55.1%)	102 (49.8%)		
IIB	68 (25.5%)	52 (25.4%)		
IIIA	7 (2.6%)	6 (2.9%)		
IIIB	20 (7.5%)	16 (7.8%)

### Fracture types

A descending order is observed in the number of cases for different fracture types: PLM-SER (335 cases, 71.0%) > PL (60 cases, 12.7%) > PLM-PER (43 cases, 9.1%) > PT (19 cases, 4.0%) > P (15 cases, 3.2%) (Table 1 and Fig. 3). According to comparison of different years, the proportion of PLM-SER is higher than that of other fracture types (Fig. 4) and a peak of incidence rate occurs in the 50–59 age group (Fig. 5). Other fracture types have no obvious age peak of incidence rate.

### Haraguchi classification of posterior malleolus fracture

The Haraguchi classification I Type, II Type, and III type of posterior malleolus fracture have 369 cases (78.2%), 49 cases (10.4%) and 54 cases (11.4%), respectively (Table 1). No statistically significant differences were observed between the two hospitals as regards the Haraguchi classification (H = 0.3, P = 0.598) (Table 3 and Fig. 3). The proportion of Haraguchi classification also shows no statistical difference (H = 2.3, P = 0.132) between the early (2014–2018) and late (2019–2022) stage groups (Table 4 and Fig. 4). According to distribution curves of age, only the Haraguchi I Type presents a significant peak in the 50–59 age group (Fig. 5).

### Tongji classification of posterior malleolus fracture

The IIA Type shows the highest number of cases (249 cases, (52.8%) for the Tongji classification of posterior malleolus fracture, followed by the IIB Type (120 cases, 25.4%), I Type (54 cases, 11.4%), IIIB Type (36 cases, 7.6%), and IIIA Type (13 cases, 2.8%) (Table 1). No statistical differences between the two hospitals were observed as regards the Tongji classification (H = 2.2, P = 0.136) (Table 3 and Fig. 3). Moreover, no statistically significant difference was observed in the proportion of Tongji classification among different years (H = 0.3, P = 0.616) (Table 4, Fig. 4). The curves in Fig. 5 indicate that the IIA Type presents an obvious peak in the 50–59 age group, while the IIB Type has a relatively flat peak.

## Discussions

The posterior malleolus is a component of the distal tibiofibular complex, and it participates in and maintains ankle joint stability [14–16]. The ankle joint bears loads of approximately 3–5 times of body weight when individuals are standing or walking with heavy loads [17, 18]. Maintaining the integrity of the ankle joint and soft tissues plays an important role in the contact area of the tibial astragaloid joint, the bearing capacity of the human body, and the stability of the ankle joint [19, 20]. If an ankle joint injury combined with posterior malleolus fracture is not treated promptly or properly, the incidence rate of traumatic arthritis may increase, thus influencing the quality of life of patients [5, 21, 22]. To increase control of the posterior malleolus fracture and improve clinical effects, the epidemiology, injury mechanism, and clinical and imaging features of posterior malleolus fracture must be explored. Nevertheless, large-scaled epidemiological surveys of posterior malleolus fracture are rare. In this study, the information of 472 inpatients for posterior malleolus fracture from Shanghai Tongji Hospital and Karamay Central Hospital were reviewed to investigate the latest epidemiological characteristics. Moreover, the injury mechanisms and morphological differences of posterior malleolus fracture in different stages (early [2014–2018] and late [2019–2022], regions (Shanghai Tongji Hospital and Karamay Central Hospital), and age groups were compared. The research conclusions herein can provide references for clinical control and relevant basic and clinical studies.

### Demographic characteristics

Chaparro et al. [23] conducted an epidemiological survey of 25 cases of posterior Pilon fracture. In their work, the average age of patients was 42 years (ranging from 22 to 62 years), 19/25 (76%) of the patients were female, and 6/25 (24%) were male. Rydberg et al. [24] performed a large-sample-sized analysis on ankle joint fracture (57,443), for which the average age of patients at injury was 55. Female patients (61%) outnumbered the male counterparts (39%). In the present study, 472 patients with posterior malleolus fracture were included, with an average age of 48.7 ± 15.6 years. Among the patients were 220 males (46.6%), with an average age of 43.3 ± 14.9 years. The rest were 252 females (53.4%), with an average age of 53.4 ± 14.7 years. The age of patients (48.7 ± 15.6) in this study was between those in the aforementioned works, and the proportion of females was higher in all three studies. However, the gender ratio slightly differed among the three studies, an outcome which might be related to the different disease types of included patients. Chaparro F et al. focused on patients with posterior Pilon fracture, Rydberg EM et al. focused on patients with ankle joint fracture, and this research focused on new patients with posterior malleolus fracture. Further study on the age distribution trend revealed that patients have an obvious peak of posterior malleolus fracture in the 50–59 age group. This finding might be related to endocrine dysfunction and the high incidence rate of osteoporosis in women after menopause.

### Comorbidities

Comorbidities influence therapeutic effect and the prognosis of patients to some extent. Qu Wenqing et al. [25] posited that the complications related to postoperative incision and bone healing increased significantly in patients with both diabetes and a history of long-term heavy smoking, which deserves special attention in ankle joint treatment. According to analysis of comorbidities in patients with posterior malleolus fracture, only 145 cases (30.7%) had comorbidities. Special attention should be paid to the comprehensive treatment of internal medicine diseases like diabetes, hypertension, and coronary heart disease during treatment of posterior malleolus fracture.

### Injury mechanism

Rydberg et al. [24] confirmed that falling is the most common injury mechanism of ankle joint fracture. Traffic accidents also account for a high proportion (29.2%), but are more likely to cause open fractures (4.7%). Moreover, males are more likely to suffer high-energy trauma. Scheer et al. [26] carried out an epidemiological survey on 673,214 patients with ankle joint fracture in emergency departments in the USA from 2012 to 2016 and revealed that the incidence rate of ankle joint fracture was 4.22/10,000 people per year. The most common injury mechanism is a fall (54.83%), followed by sports injury (20.76%), exercise injury (16.84%), jump injury (4.42%), trauma (2.84%), and others (0.30%). In the present study, low-energy injury (fall and sprain) is the main injury mechanism of posterior malleolus fracture, followed by traffic accidents and fall injury from height successively. This conclusion is consistent with those of previous reports. However, has the injury mechanism of posterior malleolus fracture changed with social development and scientific technological progresses? If so, what is the variation trend? To address these issues, the author further explored the variation trend of injury mechanism with time (early and late stages) and regions (Shanghai Tongji Hospital and Karamay Central Hospital which represent regional differences between Eastern and Western China). No obvious statistical difference was observed in the injury mechanism of posterior malleolus fracture among different years and regions. Moreover, the number of fall and sprain injury cases increases with the increase of age in adult patients and reached a peak in the 50–59 age group, followed by a progressive reduction. Traffic accidents only present a relatively flat peak in the 40–59 age group. Hence, to decrease the incidence rate of bone fracture and disability of patients, prevent fall and sprain (especially for the aged) is vital, an aim which requires the collaboration of society, family, and individuals.

### Fracture type

Posterior malleolus is an important component that participates in and maintains ankle joint stability. Pure posterior malleolus fracture is rare, and most posterior malleolus fractures are combined with internal malleolus, lateral malleolus//fibular, and tibial fractures. Posterior malleolus fractures account for approximately 7–44% of ankle joint injuries [6, 8]. The Lauge-Hansen classification [27] is one of the first ankle joint fracture classifications proposed. Ankle joint fracture is divided into four types according to positions and violence direction: supination-adduction, supination external rotation, pronation external rotation, and pronation-abduction. The supination external rotation III and IV as well as pronation external rotation IV can cause posterior malleolus fracture. Based on the injury mechanism of the ankle joint, the Lauge-Hansen classification is also one of most prevalent ankle joint fracture classification systems currently used at present. According to this classification, this work further analyzed the proportions of different ankle joint injury types involving posterior malleolus fracture and determined supination external rotation IV and pronation external rotation IV fractures. Additionally, the special fracture type (tibial shaft fracture combined with posterior malleolus fracture) is included in this research as an important component involving posterior malleolus fracture. Previous studies have rarely reported injury ratios involving posterior malleolus fracture. According to the results in this article, PLM-SER accounts for the highest proportion of posterior malleolus fracture (335 cases, 71.0%), and the same incidence trend was observed from a comparative analysis of different age groups. An obvious incidence peak occurs in the 50–59 age group. The proportions of other fracture types present a descending order: PL (60 cases, 12.7%) > PLM-PER (43 cases, 9.1%) > PT (19 cases, 4.0%) > P (15 cases, 3.2%). Further, other fracture types show no obvious peak of incidence rate. Clearly, serious trimalleolar fracture-supination external rotation is the most common fracture type once posterior malleolus fracture is developed, followed by bimalleolar fracture and trimalleolar fracture-pronation external rotation. Trimalleolar fracture is a relatively serious type of ankle joint fracture, and any improper treatment will surely influence the quality of life of patients significantly. Hence, attention must be directed toward ankle joint injury and the improvement of treatment and prognosis in clinics and in daily living.

### Haraguchi classification of posterior malleolus fracture

The Haraguchi classification, reported by Haraguchi N et al. in the Journal of Bone and Joint Surgery (American Volume) in 2006 [9], is the earliest posterior malleolus fracture classification. It covers three types: I Type: posterior lateral oblique fracture and posterolateral wedge fracture of the distal tibia, accounting for approximately 67%; II Type: internal extension type. The fracture line extends to the malleolus medialis transversely, accounting for approximately 19% of cases. This type of fracture is also called the posterior Pilon fracture; III Type: microshell type and lamellar fracture at posterior distal tibia, accounting for approximately 14%. This classification is based on the morphological features of CT scanning fracture and has been widely applied in clinics. In this work, the number of cases corresponding to the Haraguchi I, II and III Types are 369 (78.2%), 49 (10.4%), and 54 (11.4%), respectively (Table 1). According to the comparative analysis, time difference and regional difference does not influence the composition ratio of the Haraguchi classification. Further analysis of the age distribution curve reveals that only Haraguchi I type presents a significant peak in the 50–59 group. The findings of the present research are consistent with those of Haraguchi N et al. The proportion of Haraguchi I Type is highest, and those of Haraguchi II and III types are basically equivalent. This result slightly differs with the research results of Haraguchi N et al., a discrepancy which might be attributed to the inclusion of cases of tibial shaft fracture combined with posterior malleolus fracture.

### Tongji classification of posterior malleolus fracture

Treatment schemes and prognoses differ significantly given different injury mechanisms and severity. Many scholars have proposed several classification systems for posterior malleolus fracture, aiming to disclose the injury mechanism, damage mode, and damage severity, thereby guiding treatment and aiding in identifying prognosis. In 2023, Yang Yunfeng et al. [13] associated the measured value of posterior malleolus ligament structural anatomy and CT imaging features of posterior malleolus fracture based on Haraguchi and Mason classifications. They developed a new classification of posterior malleolus fracture by incorporating posterior malleolus ligament structure as well as morphological features and the injury mechanism of the posterior malleolus fragment. The author believed that this classification elaborated on the pathological anatomic feature of posterior malleolus fracture more comprehensively and that this scheme is a relatively ideal classification for the guidance of clinical diagnosis and treatment at present. The Tongji classification covers three types: I Type is fracture in the tibial attachment zone of the inferior transverse tibiofibular ligament, which is pure rotational posterior malleolus fracture. Only the inferior transverse tibiofibular ligament is pulled, thus resulting in posterior cortex avulsion fracture at the distal tibia. The II Type involves fracture in the tibial attachment zone of the inferior transverse tibiofibular ligament and posterior inferior tibiofibular ligament, and this type can be divided into the IIA and IIB Subtypes according to whether posterior malleolus fracture is accompanied by cartilago articularis and subchondral bone injuries, compression, or die-punch bone blocks. This damage is caused when the ankle joint still suffers from pure torsional violence. Torsional violence is relatively strong, resulting in fracture in the tibial attachment zone of the inferior transverse tibiofibular ligament and posterior inferior tibiofibular ligament. Ligament traction leads to a large-scaled avulsion fracture in the posterior malleolus. The IIB Type is the vertical–rotational posterior Pilon fracture. The ankle joint suffers violent-rotational composite violence. When the posterior tibial bone mass suffers a vertical load from the tali and ligament traction occurs during rotation, the posterior malleolus fracture block becomes likely to develop a proximal shift. Moreover, the tali is more likely to develop backward subluxation. The articular surfaces of the tibia and tali develop articular cartilage and subchondral bone damages and compression because of vertical violence and are often accompanied by die-punch bone blocks. The III Type involves fracture in the tibial attachment zone of the inferior transverse tibiofibular ligament, posterior inferior tibiofibular ligament, and posterior tibiotalar ligament and can be divided into IIIA and IIIB Subtypes according to the number of posterior malleolus fragments. The IIIA Subtype is the vertical posterior Pilon fracture and is caused by vertical axial violence against the posterior tibia when the ankle joint is at the falling flexion position. The cases in which posterior malleolus fragments are divided into posteromedial and posterior-lateral parts are the IIIB Type, which entails the vertical-rotational posterior Pilon fracture. This type is caused by strong vertical-rotational composite violence against the posterior tibia when the ankle joint is at the falling flexion position. The rotation of the tali causes posterior-lateral fracture at the distal tibia. Further rotation leads to posteromedial fracture at the distal tibia. With the increase of the classification level, the number of ligament structures with posterior malleolus injury increases and the fracture range expands, accompanied by increasing ankle joint instability. This development can be viewed as the intensification of damage severity. In this study, the number of IIA Type cases is highest (249 cases, 52.8%), followed by the IIB Type (120 cases, 25.4%), I Type (54 cases, 11.4%), IIIB Type (36 cases, 7.6%), and IIIA Type (13 cases, 2.8%) successively (Table 1). The composition ratios of this classification exhibit no statistical difference among different regions and age groups (Tables 3 and 4, Figs. 3 and 4). The IIA Type also presents an obvious peak in 50–59 age group. According to this classification scheme, pure rotational violence posterior malleolus fracture (IIA Type) accounts for the highest proportion and is the most common torsional violence induced large-scaled avulsion posterior malleolus fracture (involving the inferior transverse tibiofibular ligament and inferior tibiofibular ligament). The mildest damage (I Type) and the most serious damage (III Type) account for the lowest proportion in posterior malleolus fractures. In clinics, we should pay more attention to the more common II Type, explore and improve its clinical therapeutic effect, and improve prognosis. The III Type shows higher severity than other types but its incidence rate is relatively lower. Thus, comprehensive assessment and individual treatment must be combined in clinics to maximally reduce relevant complications.

### Limitations

The main shortcomings of this study are as follows. Retrospective study methods were used in data acquisition and analysis. This work neither conducted follow-up visits to treatment schemes and nor analyzed the prognosis of patients with posterior malleolus fracture. These concerns can be further discussed in depth in future research.

## Conclusions

In summary, low-energy fall and sprain is the key injury mechanism of posterior malleolus fracture. Trimalleolar fracture-supination external rotation, Haraguchi I type, and Tongji IIA type of posterior malleolus fracture are common fracture types. All these types present an obvious incidence peak in the 50–59 age group. Elderly patients have high risks of falling and experience increasing bone fragility, which are potential risk factors of posterior malleolus fracture. The early positive control has important significance. Finally, this study provides references for basic and clinical studies on posterior malleolus fracture.

## Data Availability

The data sets supporting the conclusion of this article are included in the manuscript. Upon request, raw data can be provided by the corresponding author.
